# Definite Ocular Sarcoidosis Using Endobronchial Ultrasonography with Transbronchial Needle Aspiration

**DOI:** 10.1155/2014/968697

**Published:** 2014-08-14

**Authors:** Sukhuma Warrasak, Sawang Saenghirunvattana, Ataya Euswas, Santa Methasiri, Surapon Worapongpaiboon, Supranee Nirapathpongporn

**Affiliations:** ^1^Department of Ophthalmology, Ramathibodi Hospital Faculty, Mahidol University, Rama 6 Rd., Rajthevi, Phyathai, Bangkok 10400, Thailand; ^2^Chest Unit, Samitivej Hospital, Bangkok 10110, Thailand; ^3^Bangkok Eye Center, Bangkok Hospital Medical Group, Bangkok 10110, Thailand; ^4^Pathology Unit, Samitivej Hospital, Bangkok 10110, Thailand; ^5^Radiology Unit, Samitivej Hospital, Bangkok 10110, Thailand

## Abstract

*Purpose*. To introduce a minimally invasive procedure, endobronchial ultrasound-guided transbronchial needle aspiration (EBUS-TBNA), to obtain a pathologic evidence of a definite ocular sarcoidosis in a patient who initially presented with presumed ocular sarcoidosis with pulmonary involvement. *Methods*. An EBUS-TBNA procedure was performed at perihilar lymph nodes, subcarina, and right paratrachea of the patient and specimen obtained was sent for histocytopathological studies. *Result*. Histocytopathological findings revealed aggregates of epithelioid histiocytes forming a noncaseous granuloma, a hallmark of sarcoidosis. *Conclusion*. EBUS-TBNA should be considered an alternative procedure to provide cytohistopathology proven diagnosis of definite ocular sarcoidosis.

## 1. Introduction

The diagnosis of sarcoidosis can be reliably established when there is a compatible clinical/radiological picture together with the hallmark histologic findings of noncaseating epithelioid cell granulomas. Sarcoidosis affects predominantly the lungs and thoracic lymph nodes, skin, and eyes. The most predominant finding is peribronchial thickening or bilateral lower lung involvement, especially hilar adenopathy, which occurs in patients with pulmonary sarcoidosis [1]. Ocular involvement in sarcoidosis is present in approximately 25–60% of patients with systematic sarcoidosis and may be the initial manifestation of the disease [1, 2]. Common ocular manifestations are a granulomatous anterior uveitis, posterior uveitis, or both. Posterior segment involvement including periphlebitis with perivascular sheathing, vitritis, chorioretinitis, and choroidal and optic nerve granulomas may lead to visual impairment or blindness [1]. Traditionally, transbronchial lung biopsy (TBLB) has been an approach for diagnosis of pulmonary sarcoidosis, but it can be nondiagnostic and associated with a risk of pneumothorax and bleeding [3]. In recent years, real-time EBUS-TBNA, a minimally invasive technique which used a curved linear array ultrasonic bronchoscope that provides high resolution imaging of the mediastinum using high frequency ultrasound probes attached to the tip of a flexible endoscope, allowed aspiration biopsy to obtain a definite diagnosis for malignant and benign lung lesions more safely with high diagnostic values and sensitivities compared to conventional methods which relied on a “blind” needle puncture guided by static computed tomography scans [3–5]. A systematic review and meta-analysis of EBUS-TBNA in sarcoidosis between 2004 and 2011 which included 553 patients from 15 studies revealed a diagnostic yield ranging from 54 to 93% with the pooled diagnostic accuracy being 79% (95% CI, 71–86%) and only 5 minor complications were reported [6]. This paper is the first report to demonstrate the role of EBUS-TBNA in labeling a diagnosis of definite ocular sarcoidosis in a patient who had bilateral hilar lymphadenopathy and peribronchial thickening but initially presented with presumed ocular sarcoidosis.

## 2. Case Report

**Figure 1:**
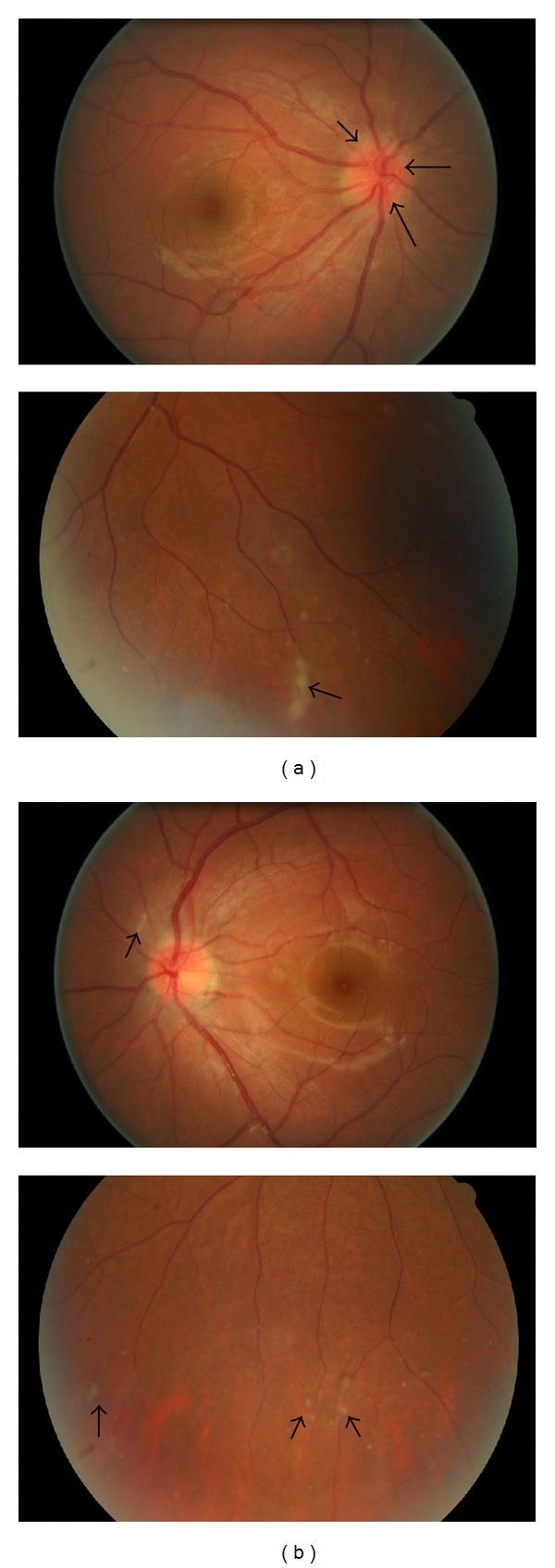
Color fundus photographs taken at initial presentation. (a) Right eye, the disc was hyperemic and its margin was slightly blurred (black arrows). Vitreous clumping and large yellow spots of chorioretinitis were seen in the inferior retina (black arrows). (b) Left eye, multiple small yellow spots of chorioretinal inflammation at the posterior pole and inferior retina (black arrows).

**Figure 2:**
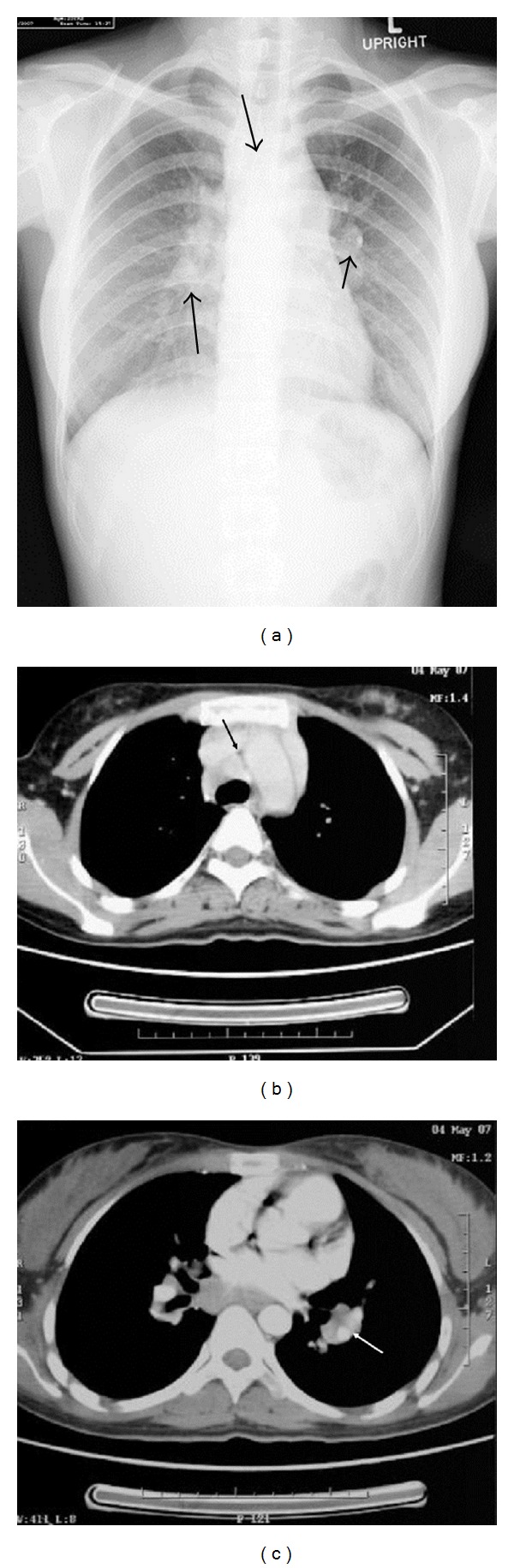
(a) Film chest PA showing widening mediastinum (5.9 cm), bilateral hilar enlargement sizes 2.1 × 6.4 cm and 2.9 × 6.2 cm. Arrows point to mediastinal nodes and both hilar nodes. The lung parenchyma was normal. (b) and (c) CT with contrast showing paratracheal nodes, subcarinal nodes, and bilateral hilar nodes.

**Figure 3:**
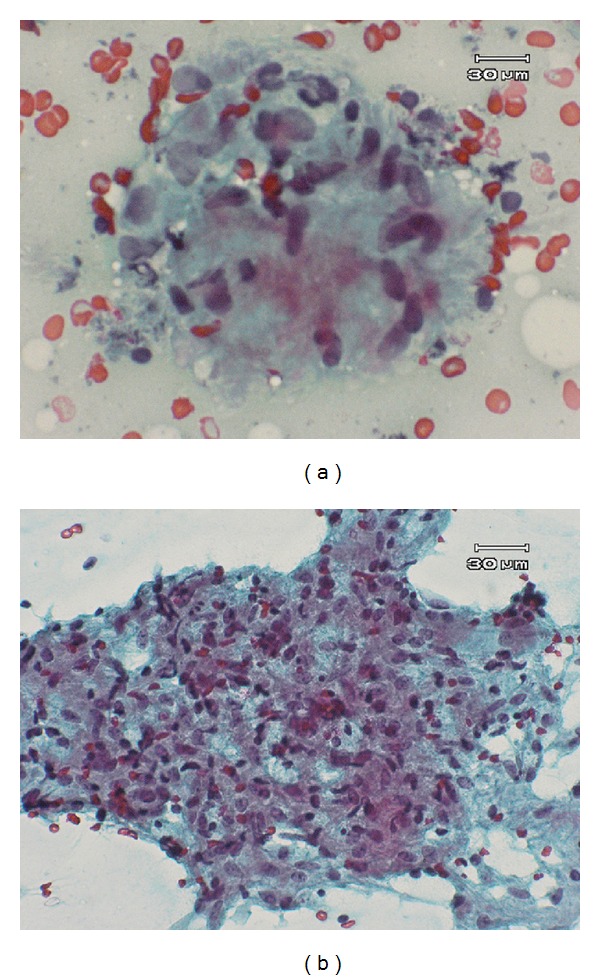
Cytohistopathological findings obtained from EBUS-TBNA specimen. (a) Noncaseating granuloma composed of epithelioid histiocytes with abundant cytoplasm (Pap stain ×400). (b) Larger aggregates of epithelioid histiocytes with admixed lymphocytes forming a large noncaseous granuloma (Pap stain ×200–400).

**Figure 4:**
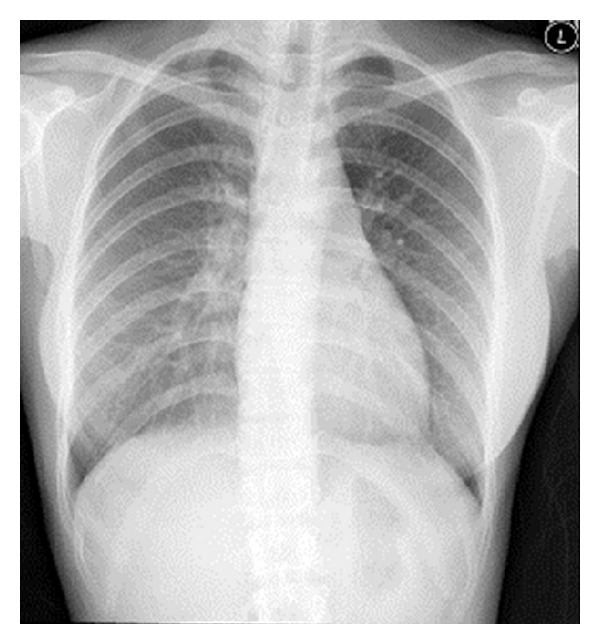
Follow-up chest film at 3 months after treatment. Mediastinum and both hilar nodes appeared normal. Note a decrease in size of superior mediastinal node (5.2 cm) and both hilar nodes (2.0 × 5.9 and 4 × 2 cm).

**Figure 5:**
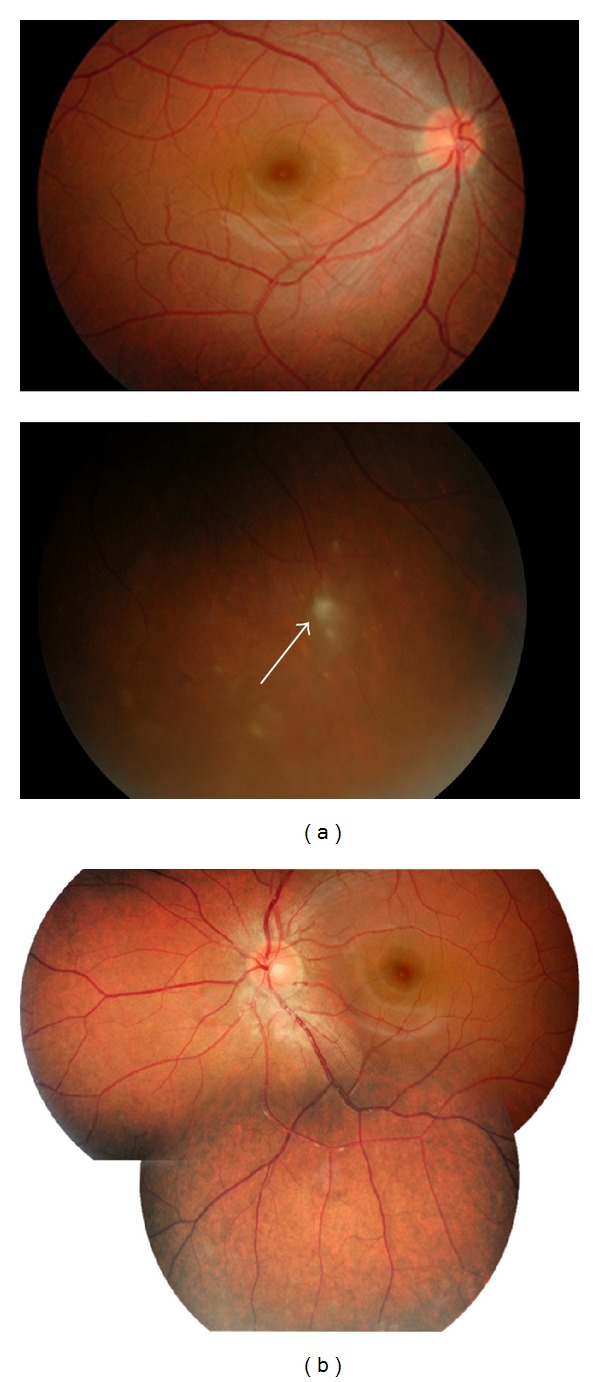
Follow-up fundus photographs taken at 3 months after treatment. Note sharp disc margin and subsiding vitreous and chorioretinal inflammation in both eyes. (a) Right eye. Thin transparent membrane covered the subsiding chorioretinal lesion (white arrow). (b) Left eye.

A 25-year-old female complained of blurring vision and noticed several small floaters in the right eye of about one month's duration. Initial ocular examination revealed best corrected distant visual acuity of 20/40 in the right eye and 20/20 in the left. Slit lamp biomicroscopy revealed fine mutton fat KPs and trace cells in the anterior chamber bilaterally. Dilated fundus examination disclosed blurred disc margin in both eyes. Multiple vitreous clumping and small yellow spots of retinitis with deposits along the venous wall were seen at the inferior retina, more intense in the right eye than in the left (Figures 1(a) and 1(b)). No external ocular involvement, that is, the conjunctiva, the episclera, the orbit, and sclera, was found, nor were there any skin lesions or palpebral lymphadenopathy. Chest X-ray PA upright (Figure 2(a)) and computerized scan of the chest (Figures 2(b) and 2(c)) demonstrated widening mediastinum with abnormal peribronchial thickening, bilateral hilar nodes enlargement, and normal lung parenchyma. Consultation to pulmonologist was made and discussion with the patient whether an attempted biopsy proven diagnosis of pulmonary sarcoidosis should be performed. The patient approved a biopsy procedure. EBUS-TBNA of the perihilar lymph nodes, subcarina, and right paratrachea was performed. The bronchoscope that was used (BF-UC160F-OL8/BF-UC260F-OL8; Olympus Medical Systems Corp., Tokyo, Japan) had an outer diameter of 6.9 mm, a 2.0 mm instrument channel, and 35-degree forward oblique-viewing optics. Scanning was performed at a frequency of 7.5 MHZ and allowed a tissue penetration of 20 to 50 mm. Image processing was performed by an endoscopic ultrasound center (EU-C60/EU-C2000; Olympus Medical Systems Corp., Tokyo, Japan). A dedicated 22-gauge needle (NA-201SX-4022 Olympus Medical Systems Corp., Tokyo, Japan) was equipped with a stylet, which was withdrawn after passing the bronchial wall, avoiding contamination during the process. Fine needle aspiration was performed by passing the needle through the airway wall and into the lymph nodes under real-time ultrasound control. Integrated color power Doppler mode was used to identify intervening vessels immediately before needle puncture. The overall operating time was 30 minutes. Cytopathology of the specimens obtained from an EBUS-TBNA of the mediastinal lymph nodes revealed microscopic aggregates of epithelioid histiocytes forming a noncaseous granuloma with a small number of reactive lymphocytes, benign bronchial epithelial cells, and few histiocytes in the background (Figures 3(a) and 3(b)). During the first 4 weeks, the patient was treated with prednisolone (Predsomed, MedicPharma, Thailand) 0.5–1 mg/kg body weight daily based on vitritis and chorioretinitis activity. The patient also received corticosteroid inhalant (Pulmicort 200 mcg/puff, Astrazeneca, Sweden) inhaled twice daily for local pulmonary treatment. For local eye treatment, the patient received prednisolone acetate 1% (Predforte, Allergan, Ireland) four times a day for 4 weeks and topical mydriatic agent, tropicamide 1% (Mydriacyl ophthalmic solution, Alcon-Couvreur, Belgium), twice daily in both eyes for 2 weeks. As posterior segment inflammation was still active but the patient could not tolerate the side effects of corticosteroid, pulmonologist decided to use another drug. Low dose cyclophosphamide (Endoxan, Dexter, Germany) in combination with low dose prednisolone 10 mg daily was given until 3 months. In a followup at 3 months, chest film (Figure 4) showed a decrease in size of superior mediastinal node (5.2 cm) and both hilar nodes (2.0 × 5.9 and 4 × 2 cm). The blurring disc margins, vitritis, and chorioretinitis improved (Figure 5). The patient was maintained with oral prednisolone 5 mg daily on alternate days until 6 months. Yearly follow-up examination for 5 visits revealed that visual acuity of 20/20 was achieved in both eyes, and the eye as well as the lung had no recurrent exacerbation.

## 3. Discussion

For patients with suspected sarcoid uveitis, rationale to use immunologic evidence to diagnose active lesion of sarcoid in the eye is limited. Though recent study reported a high CD4/CD8 ratio of lymphocytes obtained from diagnostic vitrectomy using flow cytometric analysis which delivered a high diagnostic value; such evidence provided the diagnosis of presumed ocular sarcoidosis [7]. A diagnostic vitrectomy is not a noninvasive procedure nor does it provide a direct histopathological evidence of intraocular sarcoidosis. Diagnostic yield from vitrectomy such as polymerase chain reaction (PCR) has merit in ruling out infection or for differential diagnosis of chronic endophthalmitis, intraocular lymphoma, or other chorioretinitis [8]. However, vitrectomy is more beneficial for longstanding uveitis with significant vitreous opacity, cystoid macular edema, or epiretinal membrane formation which improved visual outcome that can be anticipated following operation procedures [9, 10]. Since sarcoidosis is a multisystem granulomatous disease, establishing a diagnosis requires at least two organs for identification of involvement [11]. Clinical and cytohistopathologic findings of noncaseating granuloma obtained from EBUS-TBNA of the mediastinal nodes labeled the patient as having definite ocular sarcoidosis [12]. Despite an increasingly used technique of EBUS-TBNA in diagnosis of pulmonary mass and lymphadenopathy, small number of retrospective or a few prospective studies have addressed its role in diagnosing pulmonary sarcoidosis and none in ocular sarcoidosis [5, 6, 13]. A retrospective study compared three diagnostic modalities: EBUS-TBNA, TBLB, and bronchoalveolar lavage fluid analysis (BAL) which showed the accuracy of sarcoidosis, which is significantly better by EBUS-TBNA compared to TBLB and BAL (91.4%, *P* < 0.001) [14]. Another study compared between EBUS-TBNA and TBLB, and endobronchial biopsy (EB) demonstrated a significantly better diagnostic yield (54–93%) by EBUS-TBNA compared to TBLB or EB biopsies in patients suspicious of stage I or II sarcoidosis [15]. A recent prospective comparison of diagnostic yield (noncaseating epithelioid cell granuloma) obtained from lymph nodes aspiration for stage I and II sarcoidosis is significantly higher for EBUS-TBNA than TBLB (94% versus 37%, resp., *P* < 0.001) [16]. However, the role of EBUS-TBNA for ocular sarcoidosis requires additional study to include more patients. The merit of early diagnosis definite ocular sarcoidosis can salvage the eye from longstanding ocular complications or undesirable drug treatment complications.

## 4. Summary 

We demonstrated the role of EBUS-TBNA as an alternative procedure in labeling a diagnosis of definite ocular sarcoidosis and pulmonary sarcoidosis in a young patient who initially presented with presumed ocular sarcoidosis.
